# Flow rate dependent continuous hydrolysis of protein isolates

**DOI:** 10.1186/s13568-018-0548-9

**Published:** 2018-02-10

**Authors:** Tim Sewczyk, Marieke Hoog Antink, Michael Maas, Stephen Kroll, Sascha Beutel

**Affiliations:** 1 Institute for Technical Chemistry, Leibniz University Hannover, Callinstraße 5, 30167 Hannover, Germany; 2 Advanced Ceramics, University Bremen, Am Biologischen Garten 2, 28359 Bremen, Germany; 3 Institute for Bioplastics and Biocomposites, Hochschule Hannover, Heisterbergallee 12, 30453 Hannover, Germany

**Keywords:** Immobilization, Proteases, Bioactive peptides, Continuous process, Food protein

## Abstract

Food protein hydrolysates are often produced in unspecific industrial batch processes. The hydrolysates composition underlies process-related fluctuations and therefore the obtained peptide fingerprint and bioactive properties may vary. To overcome this obstacle and enable the production of specific hydrolysates with selected peptides, a ceramic capillary system was developed and characterized for the continuous production of a consistent peptide composition. Therefore, the protease Alcalase was immobilized on the surface of aminosilane modified yttria stabilized zirconia capillaries with a pore size of 1.5 µm. The loading capacity was 0.3 µg enzyme per mg of capillary with a residual enzyme activity of 43%. The enzyme specific peptide fingerprint produced with this proteolytic capillary reactor system correlated with the degree of hydrolysis, which can be controlled over the residence time by adjusting the flow rate. Common food proteins like casein, sunflower and lupin protein isolates were tested for continuous hydrolysis in the developed reactor system. The peptide formation was investigated by high-performance liquid chromatography. Various trends were found for the occurrence of specific peptides. Some are just intermediately occurring, while others cumulate by time. Thus, the developed continuous reactor system enables the production of specific peptides with desired bioactive properties.

## Introduction

Food proteins are important macronutrients providing the human body with essential amino acids. Nutrients of hydrolyzed products are better accessible for the human body, since the proteins are pre-digested (Koopman et al. 2009). Generally, natural enzymatic breakdown occurs in the gastrointestinal tract. Bacteria and cells release digestive enzymes or their inactive precursors. In an activated form, they hydrolyze the protein substrate at specific sites and the released peptides can be absorbed. Some polypeptides with a size of 2–20 amino acids show biological activities in terms of regulating the gastrointestinal, nervous, cardiovascular or immune system (Meisel and Bockelmann 1999; Nagpal et al. 2011; Korhonen and Pihlanto 2006) and show anti-microbial (Hancock and Sahl 2006; Haque and Chand 2008) or anti-carcinogenic properties (Fitzgerald 1998; Suarez-Jimenez et al. 2012). When using enzymes for digesting food proteins, an enzyme specific peptide composition, the so-called peptide fingerprint is formed. In chromatographic analysis the hydrolysate is separated and the peptides show a highly specific and reproducible pattern. The entirety of produced protein fragments and peptides is called proteolysome (Pimenta and Lebrun 2007).

In industrial processes, protein hydrolysis is often carried out in batch processes and is mostly used in fermentation and enzyme based processes, like brewing, cheese manufacturing, meat tenderization or baking (Godfrey and Reichelt 1982). Hydrolysis is conducted by an enzymatic or acidic breakdown of proteins (Tsugita and Scheffler 1982). For food protein hydrolysates, the substrate and enzymes are mixed in huge, tempered tanks, and require continuous stirring, as well as a constant temperature and pH level for several hours. The enzyme is then inactivated by pH-shifting and/or increasing the temperature. These processes are uncontrolled, so the resulting peptide fingerprint and the product properties vary and large quantities of expensive enzymes are used only once (Pasupuleti and Braun 2008; Hou et al. 2017).

To reduce energy and enzyme costs and produce continuously defined hydrolysates, the enzymes need to be stabilized. This can be done by protein engineering, chemical modification, immobilization or adding additives for stabilization. The most common method is to immobilize the enzymes on a solid support (Moehlenbrock and Minteer 2011). To this end, the enzymes are chemically modified by adding crosslinkers like glutaraldehyde to the peptide chain (Walt and Agayn 1994). Crosslinking can also be carried out in a two step system with reagents such as 1-ethyl-3-(3-dimethylaminopropyl) carbodiimide (EDC). To increase the efficiency of EDC-mediated amine-coupling, the carboxylates are activated by *N*-hydroxysuccinimide (NHS) which form an amine-reactive NHS-ester. The covalent amide bond is then formed with the secondary amine of the coupling enzyme, respectively the amine group of the support (Staros et al. 1986).

As a support, highly porous solids such as ceramic capillaries provide a large surface area in proportion to its volume. Using porous ceramics has several advantages compared to polymer membranes or particulate supports. They can be produced to have relatively high levels of mechanical strength, corrosion resistance and stability under high temperatures and pressures and they do not show any swelling behavior in liquid media. The pore diameter can be adapted to its purpose such as bacteria filtration (Kroll et al. 2010), oil–water separation (Zhu et al. 2016) or gas-conversion (Xue et al. 2016). However, to combine reaction and separation within the same unit, modifications of the ceramic capillary surface are necessary. Common strategies for membrane activation include chemical treatments like hydroxylation, followed by silanization with 3-aminopropyltriethoxysilane (APTES) for linking the biocatalyst (Kroll et al. 2012). Since the enzymes are covalently bound to the amino group of the support, these reactors allow high recovery in enzyme activity. Most applications of immobilized enzymes in industry are based on a conversion of sugar (Jensen and Rugh 1987; Bhosale et al. 1996) or oil (Noureddini et al. 2005; Tan et al. 2010), but rarely protein.

To enable and characterize a continuous protein hydrolysis for the production of bioactive peptides under defined conditions, several natural proteins, that are frequently used as food proteins were chosen as models for investigating hydrolysis. These were lupin protein, sunflower protein and casein. A ceramic capillary module is described and characterized for continuous food protein proteolysis and defined systematic proteolysome mapping. Therefore, proteases have been immobilized onto the macro porous ceramic support. By operating the system as a continuous reactor, the degree of hydrolysis and peptide formation is directly related to the residence time. Thus, also intermediate stages of proteins and peptides that are formed temporarily can be detected by altering the flow rate.

## Materials and methods

### Materials

Four different proteolytic enzymes were tested for their activity against the food protein substrates (Table 1). All enzymes, proteins and reagents were obtained from commercial sources and used without further purification. The model protein casein was purchased from Sigma Aldrich, USA and the lupin and sunflower protein isolates were purchased from Vegan Fitness & Food, Germany. The chemical reagents such as 4-(2-aminoethyl) benzenesulfonyl fluoride hydrochloride (AEBSF) protease inhibition reagent Pefabloc SC, 3-aminopropyltriethoxysilane (APTES, 99%), *N*-hydroxysuccinimide (NHS), *N*-(3-dimethylaminopropyl)-*N*′-ethylcarbodiimide hydrochloride (EDC) were obtained from Sigma Aldrich, USA. The Coomassie Brilliant Blue G250 was obtained from Serva Electrophoresis, Germany. The yttria (3%) stabilized zirconia (TZ-3Y-E) was obtained from Tosoh, Japan. Ammonium sulphate, methanol, phosphoric acid, MES and Tris buffer were purchased from Carl Roth, Germany.

**Table 1:** Proteolytic enzymes for protein hydrolysis

Enzyme	E.C. number	Specific activity (U/mg)	Supplier
Alcalase^®^ 2.5 FG	3.4.21.62	1.375^a^	Novozymes, Denmark
Subtilisin A	3.4.21.62	7–15	Sigma Aldrich, USA
α-Chymotrypsin	3.4.21.1	40	Sigma Aldrich, USA
Trypsin	3.4.21.4	40	Sigma Aldrich, USA

^a^According to King and Moss (1963)

### Enzyme immobilization

For coupling the enzyme with the APTES-linker, a slightly modified protocol according to Hermanson (2013) was applied. A 0.1 M MES buffer with 0.5 M NaCl and 0.01 M CaCl at pH 6 was used for dissolving 10 mM NHS and 20 mM EDC. After 10 min of equilibration, 0.5 g/L of enzyme was added to the solution. The ceramic capillary was then added for immobilizing the enzymes and was gently rotated on a Tube Rotator (PTR-35, Grant, UK) at 4 °C over night. Before using the capillary for continuous hydrolysis, it was rinsed and flushed with 5 mL of protein buffer at a flow rate of 400 µL/min.

### Protein feed solutions

For the protein feed used in the capillary module, several natural protein sources have been selected. Casein is a well described protein that is obtained from mammalian milk. It is the main component of bovine milk and consists of smaller phosphoproteins (< 25 kDa), αS1, αS2, β, κ and γ, which form micellar structures with reduced water solubility. To increase solubility, 5 g/L of casein were dissolved in 0.05 M Tris buffer at pH 7.8. The mixture was then stirred and heated to 80 °C for 15 min.

Most plant seed storage proteins are composed of globulins (11S and 7S) and albumins (2S). The albumins are water-soluble, whereas the globulins form large hexameric structures. Lupin seed protein (*Lupinus albus*) mainly consists of α- (11S), β- (7S), γ- (7S) and δ-conglutin (2S), with a content of more than 35% of α- and 45% of β-conglutin (Duranti et al. 2008). The sunflower seed protein (*Helianthus anuus*) is lacking the 7S globulin and consists of α-, α′-, β-helianthinin (11S) and 2S albumin, with a content of more than 50–70% of helianthinin (Žilic et al. 2010). In order to increase solubility of lupin and sun flower protein, 4 g/L were each dissolved in 0.1 M Tris buffer at pH 12. The mixture was stirred over night at 4 °C and was adjusted to pH 7.8 the next day. The solution was then stirred for 1 h at room temperature. Before the protein solution for continuous hydrolysis was used, the solutions were filtered with a 0.45 µm PES membrane (Wicom, Germany).

### Experimental setup

**Fig. 1:**
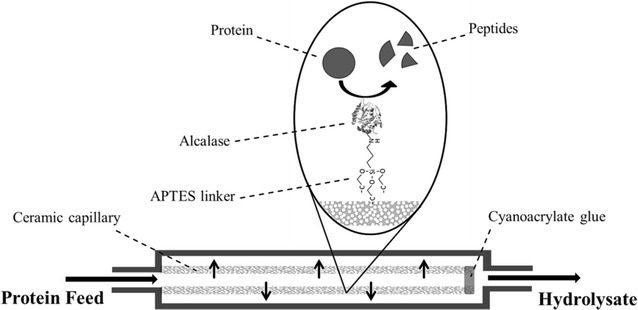
Capillary module. Enzymes are immobilized onto the APTES functionalized ceramic support to enable a continuous hydrolysis of the protein

The basic setup of the continuous reactor consists of a single enzyme loaded ceramic capillary, made from yttrium stabilized zirconia that is fixed in a custom designed stainless steel housing. The system can be considered as a PFR (plug flow reactor system) to define basic characteristics. The protein solution is pumped through the capillary module by a peristaltic pump (IPC, Ismatec, Germany). The whole capillary module is embedded in a column oven (Techlab, Germany), so the temperature can be adjusted to 37 °C. The capillary end is sealed with cyanoacrylate glue for forcing the flow from the intracapillary space (ICS) to the extracapillary space (ECS). The enzyme is immobilized on the activated surface of the ceramic capillary by an APTES linker (Fig. 1) and is reached by the protein feed through the forced convective flow through the capillary pores. The immobilization process allows the complete utilization of the available surface of the capillary. This means that the enzymes can be immobilized on the interior and exterior surface as well as the pore walls. The capillary has an average pore size of 1.5 µm and a length of 10 cm with an outer diameter of 1.8 mm and an inner diameter of 1 mm. For each experiment the ceramic capillary is replaced with a new enzyme immobilized one, to prevent protein contaminations.

### Characterization methods

#### Enzyme quantification

The enzyme concentration was determined using the Bradford reagent RotiQuant (Carl Roth, Germany) according to the manufacturer’s protocol. To determine the amount of immobilized enzyme the difference between initial and remaining enzyme concentration of the supernatant was calculated and referred to enzyme loaded onto the capillary. The amount of washed off enzyme was negligible.

#### Determination of immobilized enzyme activity

In order to determine the enzyme activity, the conversion of Boc-l-alanine-4-nitrophenyl ester (Sigma Aldrich, USA) to 4-nitrophenol was quantified. A stock solution was prepared with 16 mM Boc-alanine-4-nitrophenyl ester dissolved in 80% acetonitrile. For enzyme preparation, 50 mg YSZ powder (yttrium stabilized zirconia) were immobilized according to the protocol (see “Enzyme immobilization”) and washed three times with buffer. The powder was stirred in a beaker with 20 mL 0.1 M Tris Buffer at pH 7.8. In the following step, 200 µL of stock solution were added to the glass beaker to set up a final concentration of 160 µM. The solution was run at 1 mL/min through a low volume flow-through cuvette (Hellma Analytics, Germany) and adsorption was measured at 405 nm using a UV–VIS spectrophotometer (Genesys 10S, ThermoFisher, USA). To avoid ZrO_2_ particle interference, a low volume 0.45 µm PES filter (Wicom, Germany) was used before the solution was pumped to the spectrophotometer. To compare the activity of immobilized and native enzyme, an equivalent quantity of native enzyme was used in the setup and the slopes of the linear graphs were compared. All measurements were made in triplicates.

#### Proteolytic batch digestion

All batch digestions were carried out by setting up the protein solution with an enzyme concentration of 1 mg/L. The samples were incubated in a water bath at 37 °C for 30 min. After incubation the enzymes were inhibited using 1 mM AEBSF and the samples were kept on ice.

#### HPLC analysis of protein hydrolysate

The hydrolysate samples were analyzed using an HPLC (Chromaster, Hitachi, Japan). Separation was achieved on a reversed phase Aeris Peptide 3.6 µm XB-C18, 250 × 4.6 mm (Phenomenex, USA) with an Ultra Cartridge C18-Peptide Security Guard column (Phenomenex, USA). The mobile phase eluent buffer A consisted of 94.9% water, 5% acetonitrile and 0.1% trifluoricacetic acid. Eluent B consisted of 19.9% water, 80% acetonitrile and 0.1% trifluoricacetic acid. The gradient elution was carried out using the following timetable: from 0% B to 15% in 10 min, to 35% in 20 min, to 60% in 10 min, to 100% in 3 min, maintaining 100% for 3 min, to 15% in 0.1 min, keeping 15% for 6 min. The injection volume was 10 µL and the flow rate was 400 µL/min at 40 °C. The analytical wavelength was 214 nm and as a standard the HPLC peptide standard mixture (Sigma Aldrich, USA) was used.

#### SDS-PAGE

All gel electrophoretic analyses were performed under non-reducing conditions with a 12% Tris gel using the Bio-Rad Protean System (Bio-Rad, USA) according to the manufacturer’s manual. As protein ladder a 10–250 kDa prestained marker was used (ThermoFisher, USA). Protein staining was performed with Coomassie blue according to Fairbanks et al. (1971).

## Results

### Batch proteolysis and protease screening

**Fig. 2:**
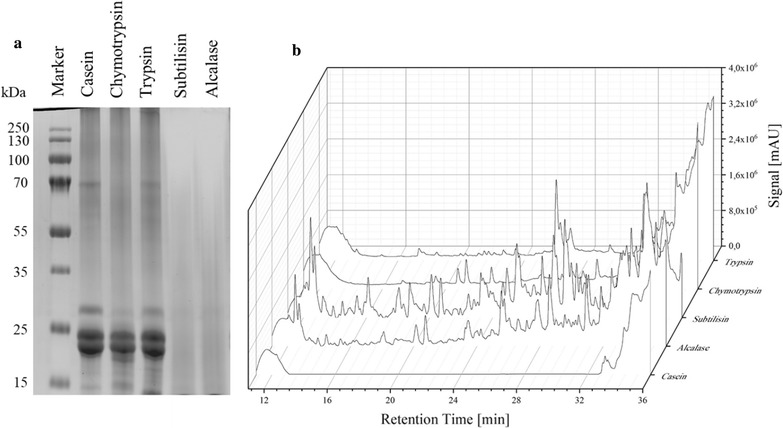
Proteolytic batch digestion of casein by different proteases. **a** Non-reduced SDS-PAGE 12% Tris gel stained with Coomassie Brilliant Blue R-250, molecular mass standard Prestained Protein Marker (250 kDa), **b** HPLC analysis performed on reversed phase C18 column

In order to assess the proteolytic activity of various serine proteases, batch digestions were carried out with casein as a model protein. An enzyme, creating a high degree of hydrolysis in short time, was required for the use in the continuous reactor system. Therefore, digestive enzymes such as trypsin and α-chymotrypsin and subtilisin originated from *Bacillus licheniformis*, respectively Alcalase 2.5, were used. An amount of 1 mg/L enzyme was used for each reaction mixture and the hydrolysates were then analyzed by SDS-PAGE and HPLC. The protein bands during digestion with subtilisin or Alcalase completely disappeared, whereas casein digested with trypsin and chymotrypsin remained almost unaffected (Fig. 2a). The enzymatic breakdown into smaller protein fragments and peptides was then verified by HPLC analysis. The number of peaks generated in subtilisin and Alcalase casein hydrolysates was significantly higher compared to trypsin and chymotrypsin. The subtilisin-family enzymes showed some similarities in their peptide fingerprint (Fig. 2b).

**Fig. 3:**
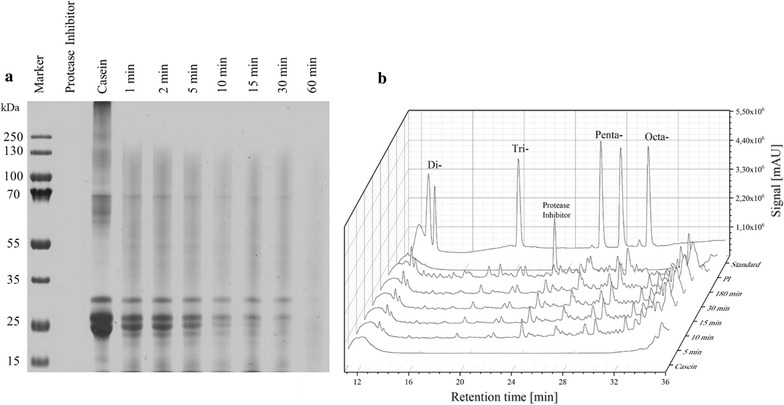
Casein digestion with Alcalase over time. **a** Non-reduced SDS-PAGE 12% Tris gel stained with Coomassie Brilliant Blue R-250, molecular mass standard Prestained Protein Marker (250 kDa), **b** HPLC analysis performed on reversed phase C18 column, using HPLC peptide standard mixture

When digesting casein with a protease like Alcalase, the peptide formation occured within a few minutes and most casein protein components were digested after 30–60 min (Fig. 3a). Even though Alcalase is regarded as an unspecific protease, the peptide fingerprint is highly specific. Some peptide peaks are formed in the very first seconds of digestion, others are formed in the later process or are just intermediately occurring (Fig. 3b).

### Setup and evaluation of the continuous digestion system

The substrates residence time is found to be inversely proportional to the flow rate. The residence time is doubled by halving the flow rate. For the conversion of a specific substrate correlating results are obtained. Here the conversion of the nitrophenyl ester to 4-nitrophenol shows, that also the product formation is inversely proportional to the flow rate. When the flow rate is reduced to its half, the formed product is doubled in the same time (Fig. 5a). For continuous hydrolysis it can therefore be said, that the higher the residence time of the substrate in the enzyme loaded capillary, the more product is formed continuously. Similar results are obtained for the continuous hydrolysis of protein substrates. The enzyme loading capacity is determined with 0.3 µg per mg of capillary. The average weight of a capillary is 550 mg, so 165 µg of Alcalase can be immobilized on the specific surface of a capillary. The enzyme activity is measured with the described method and shows a residual enzyme activity of 43%.

**Fig. 4:**
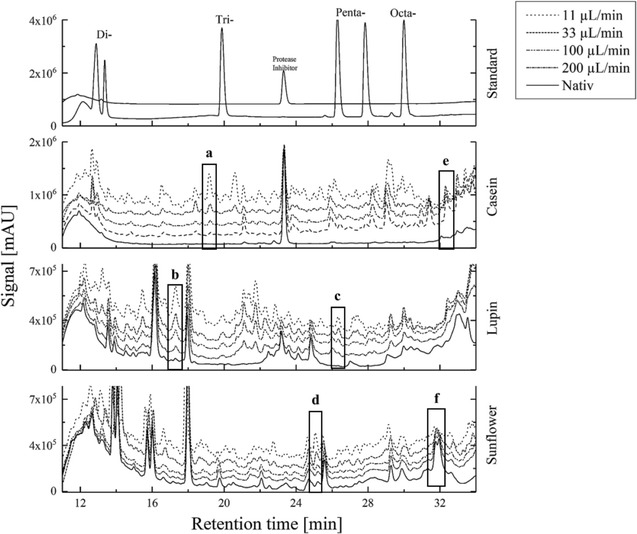
Flow rate dependent protein hydrolysis of various food proteins. Increasing peak intensities with lower flow rates (a–d), peaks intensified at specific flow rates (e) and peaks decreasing with lower flow rates (f)

When continuously digesting food proteins such as casein, lupin and sunflower protein, the formation and intensity of most peptide peaks is flow rate dependent or more specifically it is dependent on its residence time (Fig. 4). A significant and visible peptide fingerprint formation was observed at a flow rate of 200 µL/min and lower. Reducing the flow rate further increases peak formation and significantly intensifies the peaks (a–d). Some peaks are decreasing at slower flow rates (e).

**Fig. 5:**
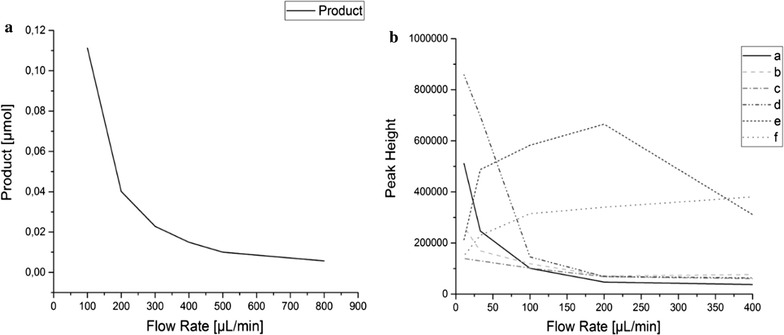
Flow rate dependent product formation. **a** Flow rate dependent formation of nitrophenol by converting the Boc-l-alanine-4-nitrophenyl ester, **b** flow rate dependent formation of peptide peaks

In the peptide formation, specific peaks increased in their peak height (a–d) while others decreased or showed a negative correlation (e–f). In particular, peak f is formed at flow rates of 400 µL/min, but is less intense at lower flow rates. Peak e is predominantly occuring at flow rates of 200 µL/min (Fig. 5b).

**Fig. 6:**
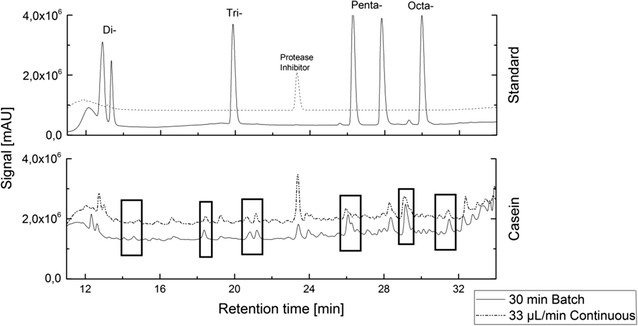
Comparison of batch and continuous hydrolysis. Protein hydrolysates of native and immobilized enzymes show a similar peptide fingerprint

For a comparison of batch- and continuous proteolysis, the residence time in the continuous reactor can be directly compared with the same batch processing time. Thus, the fingerprint formed with a continuous flow rate of 33 µL/min, providing a residence time of 30 min, can be directly compared with a 30 min batch digestion (Fig. 6). As can be seen, the peptide fingerprint shows various similarities although in general more short peptide peaks with lengths of two and three amino acids are found in the batch digestion. The similarities in the peptide pattern in the area of the four- and five-chain peptides are larger than for two to three-chain short-chained peptides. In this way, each flow rate dependent peptide fingerprint resembles a defined proteolysis time in batch processing and, most important, the consistency of the produced peptide fingerprint is stationary over time. This enables the production of specific peptide fingerprints in the developed continuous reactor system.

## Discussion

This work enables a flow rate dependent and defined production of protein hydrolysates using immobilized enzymes. A continuous reactor system with a ceramic capillary module was developed and tested with various combinations of enzymes and model protein substrates. In comparison of batch and continuous hydrolysis, more dipeptides are formed in the batch hydrolysis, but similarities in peptide size are found in the range of tri- and octapeptides. As described by Guisán et al. (1997) immobilized enzymes underlie a steric hindrance. Since the enzymes were successfully immobilized with APTES linkers, other silanes with longer spacer arms and surface modifications could be an option for further improvement of the enzyme activity. Also, the module can be easily adapted to its purpose by variation of pore size, capillary length or number. Especially, the number of capillaries can be increased to 30 or more capillaries in a multi capillary module for scaling up and thus increasing the protein feeds flow rate. Pretreating the protein feed solution as described in the method section, was feasible for the use in a microporous reactor system. Adding an ultrasonication pretreatment step to reduce protein aggregation could be helpful for less soluble proteins (Lee et al. 2016). Furthermore, immobilizing other proteases and applying complex protein feeds, such as blood sera for continuously digesting globulins, could be also promising targets as preliminary experiments show.

Even though, the idea of a continuous protein hydrolysis is old and had its first peak in the early 70 s. At that time, protein sources such as fish and soy protein were used as a substrate (Adler-Nissen 1976, 1978; Cheftel et al. 1971). In the 1990s, the focus was placed on the continuous hydrolysis of milk derived proteins (Mannheim and Cheryan 1990; Perea and Ugalde 1996). Until today no approach of continuous protein hydrolysis is found in industry (De Gonzalo and Dominguez de María 2017). Most products made from protein hydrolysates are used for animal nutrition (Hou et al. 2017) or as a food supplement for infants. Initial attempts have been carried out to reduce the allergenic potential for infants by utilizing immobilized enzymes (Pessato et al. 2016). Bioactive peptides are mainly found in functional foods, most of which are fermented dairy products (Dullius et al. 2018; Hafeez et al. 2014). Medical applications or treatment approaches using bioactive peptides on a large scale are still rare. So far, very little is known about the flow rate dependent hydrolysis and their effect on the peptide fingerprint and its peptide composition. Therefore, the approach of flow rate dependent hydrolysis is particularly suitable for the production of defined peptides patterns. As the results demonstrate, specific peptide peaks are formed at a defined flow rate, respectively the proteins residence time. In this case, high flow rates would enable a constant low degree of hydrolysis, creating macromolecular peptides. As shown by Lumen (2005) even macromolecular peptides with more than 43 amino acids can have beneficial bioactive properties. By production of defined peptide fingerprints in the continuous reactor system specific peptides can be produced, isolated and screened for their bioactive properties. This enables the systematic investigation of the proteolysome of different proteins and its impact on the human metabolism.

For the development of large-scale processes and the production of specific bioactive peptides in the relatively new field of therapeutic peptides, the module-based approach of continuous reactors is also suitable for reducing energy-intensive process steps such as heat inactivation and furthermore the reduction of chemical wastes such as acids. Furthermore, the immobilized enzymes can be reused and the production time can be reduced by scaling up the process, since a flow rate of 11 µL/min/capillary resembles a residence time of more than 90 min. In the future, further stability and long-term tests could be carried out under flow conditions.

## Abbreviations

AEBSF: 4-(2-aminoethyl) benzenesulfonyl fluoride hydrochloride
APTES: 3-aminopropyltriethoxysilane
CaCl: calcium chloride
ECS: extracapillary space
EDC: *N*-(3-dimethylaminopropyl)-*N*′-ethylcarbodiimide hydrochloride
HPLC: high performance liquid chromatography
ICS: intracapillary space
MES: 2-(*N*-morpholino)ethanesulfonic acid
NaCl: sodium chloride
NHS: *N*-hydroxysuccinimide
PES: polyethersulfone
PFR: plug flow reactor
SDS-PAGE: sodium dodecyl sulfate-polyacrylamide gel electrophoresis
YSZ: yttrium stabilized zirconia
